# Correction: Breastfeeding transition in Oman: A generation shift or a product of social development? A qualitative study on three generations of Omani mothers

**DOI:** 10.1371/journal.pone.0326151

**Published:** 2025-06-10

**Authors:** The PLOS One Staff

## Notice of republication

This article was republished on May 23, 2025, to correct error in the reference 29 that was introduced during the typesetting process. The publisher apologizes for this error. Please download the article again to view the corrected version. Both the originally published, uncorrected article and the republished, corrected article are provided here for reference.

## Supporting information

S1 File Originally published, uncorrected article. (PDF)

S2 File Republished, corrected article. (PDF)
