# Supplementary material for: Correction: Breastfeeding transition in Oman: A generation shift or a product of social development? A qualitative study on three generations of Omani mothers
Source: PLoS One. 2025 Jun 10;20(6):e0326151. doi: 10.1371/journal.pone.0326151 (PMC12151396; doi:10.1371/journal.pone.0326151)
Supplement: S2 File — (PDF) [file pone.0326151.s002.pdf]

PLOS ONE

RESEARCH ARTICLE

Breastfeeding transition in Oman: A generation shift or a product of social development? A qualitative study on three generations of Omani mothers

Atika Khalaf[1,2], **Rebecca Mathew**[3], **Pernilla Ny**[4]*

1 The PRO-CARE Group, Faculty of Health Science, Kristianstad University, Kristianstad, Sweden, **2** Hind Bint Maktoum College of Nursing and Midwifery, Mohammed Bin Rashid University of Medicine and Health Sciences, Dubai, UAE, **3** Department of Nursing, Fatima College of Health Sciences, Ajman, United Arab Emirates, **4** Department of Health Sciences, Midwifery Research, Reproductive, Perinatal and Sexual Health, Faculty of Medicine, Lund University, Lund, Sweden

* pernilla.ny@med.lu.se

OPEN ACCESS

Citation: Khalaf A, Mathew R, Ny P (2025) Breastfeeding transition in Oman: A generation shift or a product of social development? A qualitative study on three generations of Omani mothers. PLoS One 20(4): e0319789. https://doi.org/10.1371/journal.pone.0319789

Editor: Habiba I. Ali, United Arab Emirates University, UNITED ARAB EMIRATES

Received: May 17, 2024

Accepted: February 9, 2025

Published: April 17, 2025

Copyright: © 2025 Khalaf et al. This is an open access article distributed under the terms of the Creative Commons Attribution License, which permits unrestricted use, distribution, and reproduction in any medium, provided the original author and source are credited.

Data availability statement: The data used in our study contain potentially identifying or sensitive patient information and can therefore not be shared as a whole set, not even in a de-identified form. The restrictions are imposed by the IRB at Sultan Qaboos University and the Ethical Board at the Ministry of Health. The data can be requested from the Ethical Board in the College of Nursing at Sultan Qaboos University (https://www.squ.edu.om/nursing), PO Box

Abstract

Background

Exclusive breastfeeding is essential for infant health, yet its practice varies across generations and cultures. Although the health benefits of breastfeeding have been well known for decades, the utilization of infant formula feeding worldwide and in Oman, in particular, continues to rise, most likely as a result of a lack of social support and the time limit faced by working mothers. Little is known about the factors affecting Omani mothers' breastfeeding experiences and practices. Therefore, this study aims to gain an in-depth understanding of the experiences of breastfeeding practices among three generations of Omani mothers.

Methods

This qualitative study investigated exclusive breastfeeding practices among three generations of Omani mothers. Participants were recruited using a selective approach followed by a snowball technique, resulting in a total of 50 women, which included 17 first-generation mothers, 18 grandmothers, and 15 great-grandmothers. Participants were characterized by varying ages and socio-demographic backgrounds. Semi-structured face-to-face interviews were conducted in Arabic, and data collection continued until data saturation was reached. Data were analyzed using qualitative content analysis methodology, ensuring robustness and credibility.

Results

The analysis yielded two main categories reflecting the mothers' experiences, three representing the grandmothers' experiences, and two concerning the great-grandmothers' experiences. While overarching themes like the significance of breastfeeding emerged across all generations, disparities were seen concerning challenges, beliefs, and support systems. Mothers highlighted contemporary hurdles such as work-life balance and societal

66, Al-Khoudh 123, Muscat, Oman or from Omar Alzaabi, Assistant Dean for Postgraduate Studies and Research, College of Nursing (alzaabi@squ.edu.om).

Funding: AK recieved funds from the Deanship Funds at the College of Nursing at Sultan Qaboos University, Muscat, Oman (RF/CON/MCHH/22/01). URL of the funder website is https://www.squ.edu.om/research/Deanship-of-Research/About-the-Deanship. The funders had no role in study design, data collection and analysis, decision to publish, or preparation of the manuscript.

Competing interests: The authors have declared that no competing interests exist.

pressures, whereas grandmothers emphasized inherited practices and cultural norms. Great-grandmothers reflected on the evolution of societal and familial dynamics impacting breastfeeding traditions. Despite these differences, a shared commitment to breastfeeding and recognition of its importance for maternal and child well-being was evident across generations.

Conclusion

The findings underscore the importance of societal support, healthcare provider education, and workplace policies in promoting exclusive breastfeeding. Targeted interventions are needed to address barriers to breastfeeding and empower women to make informed feeding choices. By addressing these challenges, societal institutions can contribute to achieving higher rates of exclusive breastfeeding and improve maternal and child health outcomes in Oman.

Introduction

Exclusive breastfeeding (EBF) is recommended for the first six months of the child's age [1] as it is one of the most effective methods to protect the child's and mother's health [2]. Children should be breastfed on demand, day and night, without the competition of bottles, teats, or pacifiers. In addition, from the age of 6 months, adequate complementary foods should be introduced, while breastfeeding continues up to at least 2 years of age [1]. However, this is not the case in many countries [3], including those previously known with a high prevalence of EBF, such as Oman.

In Oman, the rate of EBF for the first six months of life declined from 32.8% in 2014 to 23.2% in 2017 [4]. As a high-income country, Oman belongs to the countries with the highest rates of children ever breastfed, with the number being 98% [2]. Although the number of infants who are breastfed within the first hour after birth is 71.1% in Oman and 72.8% are breastfed for 9 to 11 months, it is reported that 50.7% of babies are exposed to bottle-feeding [5], which indicates that the number of babies who are exclusively breastfed is low [5]. Oman's Ministry of Health (MOH) reported a decline in mothers beginning EBF after delivery from 93.4% in 2015 to 90.8% in 2018; however, this percentage dropped significantly to 8.7% of children exclusively breastfed at the age of six months at the same period due to the gradual introduction of foods [6].

Moreover, a recent study of the perception of Omani women of EBF [7] showed that women's ability to breastfeed relies on their adaptability and resistance to breastfeeding challenges. Additionally, motivations for EBF such as the belief that it contributes to the health of their children, is convenient and easily accessible, and is supported by their families were reported [8]. Barriers to EBF included concerns about insufficient milk, maternal employment, and limited maternity leave [7,8]. Mothers' good knowledge and positive attitudes about breastfeeding play key roles in the process of EBF practices [9]. To enhance maternal attitudes and raise their knowledge about healthy breastfeeding practices, it is crucial to shed light on the current practices and the perceptions of these from the perspectives of the mothers. In addition, lessons can be learned from different generations and their breastfeeding practices [10].

The generational shift including the involvement of women in the labour force in combination with the limited maternity leaves might barrier healthy practices and duration of breastfeeding [11]. However, the mothers' perspectives and practices of breastfeeding have never been investigated qualitatively in Oman to shed light on and give a nuanced understanding of the phenomenon, which calls for the urgent need for investigation of the current situation. The

generational influences on breastfeeding practices could help in understanding the decline in EBF among mothers in current days. Furthermore, due to the benefits of natural breastfeeding being unattainable by formula, the low rate of infants currently being exclusively breastfed during their first six months in life certainly calls for raised awareness amongst Omani parents.

As a country with a high rate of EBF [6], the breastfeeding practices in Oman can offer insights within similar contexts, shedding light on the complex interplay between cultural, economic, and social factors influencing infant feeding choices. Understanding these dynamics is crucial not only for improving maternal and child health outcomes in Oman, but also for informing strategies to promote breastfeeding worldwide. This study aims to explore exclusive breastfeeding practices among three generations of Omani mothers to gain insights into the factors influencing breastfeeding behaviours and to inform targeted interventions aimed at promoting breastfeeding practices in Oman.

Materials and methods

Study participants

In this qualitative interview-based study, the participants were Omani mothers (first-generation mothers), grandmothers (second-generation), and great-grandmothers (third-generation) recruited through a snowball technique. The following inclusion criteria were applied for first-generation mothers: 1) having given birth to a single, full-term, healthy baby within the last 12 months, 2) breastfeeding their babies, and 3) Arabic speakers. For grandmothers and great-grandmothers, the inclusion criteria were: 1) exclusively breastfed at least one of their children, and 2) were Arabic speakers. The only exclusion criterion was mothers having difficulties communicating verbally.

Sample

A total of 50 women, comprising 17 first-generation mothers, 18 grandmothers, and 15 great-grandmothers participated in the study. The participating women were characterized by varying ages and socio-demographic backgrounds as shown in Table 1, with a mean age of 33 years for mothers, ranging from 27 to 39 years, 43 years for grandmothers, ranging from 40 to 49 years, and 62 years for great-grandmothers, ranging from 54 to 73.

Table 1. Participant characteristics.

Character	Mothers (n= 17)	Grandmothers (n= 18)	Great-grandmothers (n= 15)
**Age (years), mean (min-max)**	33 (27-39)	43 (40-49)	62 (54-73)
**Number of children, mean (min-max)**	3 (1-5)	5 (3-10)	8 (3-12)
**Number of mothers who EBF their children**			
All children EBF	9	10	9
Mixed feedings	8	8	6
**Educational level**			
Primary or less		3	10
Secondary	1	6	3
Higher education	16	9	2
**Employment status**			
Employed	13	9	1
Jobseeker	3		
Housewife	1	8	14

https://doi.org/10.1371/journal.pone.0319789.t001

Sampling technique

The first participating mothers, either colleagues or relatives to the first author or the research assistant (RA), were screened for eligibility before contact was established by the RA to invite them to participate in this study. We then applied a snowball technique to recruit the remaining participants, who were given written information about the study, including contact information to the principal investigator (PI) for any queries. A selective sampling technique followed by a snowball technique is a widely recognized approach in qualitative research [12] for recruiting participants who possess specific knowledge or experiences that are related to the study's main objectives.

Study setting

Oman, officially known as the Sultanate of Oman, is administratively divided into eleven governorates (Muhafazah), which include Muscat, Dhofar, and A'Dahirah, in which Wilayat of Ibri is located, among others. Each governorate is further subdivided into districts, known as Wilayats, which are overseen by governors (Walis) responsible for local governance and dispute resolution. This administrative structure facilitates the management of Oman's diverse regions with its various cultural and geographical landscapes, contributing to the overall governance and development of the Sultanate [13].

The participants were mothers from Muscat Governorate and Wilayat of Ibri. As Muscat governorate is the capital of the Sultanate of Oman and the most densely populated governorate, and Ibri is one of the largest states in the northeastern part of the Sultanate, many of the participating mothers originated from different parts of the Sultanate, adding to the variation of the breastfeeding experiences and practices applied in different geographical areas.

Ethical considerations

Ethical clearance was obtained from the Ethical Board in the College of Nursing at Sultan Qaboos University (Ref. No. CON/DRF/2021/1), and the Ethical Board at the Ministry of Health (MH/DGSH/DG/22). A written consent form was signed by the participants at the time of the interviews after providing verbal and written information as well as time and opportunity to get answers to all questions related to the research project. The participating women consented to the recording of the interviews, the voluntary nature of their participation, the described confidentiality measures, the use of the collected data, and the right to withdraw from the study without explanation. Further, the Helsinki Declaration [14] for research in human subjects guided the data collection, ethical considerations, and data dissemination. None of the participating women was related to one another; therefore, no connections were established between the participants in the study. Every woman gave her voice and shared her unique experiences and narratives on her breastfeeding practices. Furthermore, confidentiality was guaranteed through the deidentification of the interviews, i.e., only numerical codes represented the narratives and citations given in the manuscript beside the first letter of their category, namely, M for mothers, GM for grandmothers, and GGM for great-grandmothers. In addition, both the audio records and the transcriptions in their original form were only accessed by the PI.

Data collection

An in-depth semi-structured face-to-face individual interview approach was implemented to collect data for this study. We used an interview guide developed by the research team after an extensive literature review. The interview guide comprised seven main open-ended questions about the women's exclusive breastfeeding practices, obstacles they faced during their

breastfeeding journey, facilitating factors, and their view of the current generations' exclusive breastfeeding practices compared to earlier generations.

Data were collected between September and December 2022. A trained RA, an experienced midwife, was recruited and compensated to distribute the information and consent forms, recruit participants, conduct interviews, and transcribe the data concurrently. The RA received training in obtaining informed consent, recruiting participants using a snowball sampling technique, and conducting interviews. Mothers who met the inclusion criteria and agreed to participate gave their consent before agreeing on a suitable place and time for the interview. Most of the mothers opted to be interviewed in their homes, while some preferred to be interviewed in a private office at the hospital or healthcare center where the RA recruited them. At the interview occasion, participants were given oral information about the study again and the objectives of the interviews. They were also reminded of the freedom to decline participation or withdraw from the study at any time.

The data collection continued till data saturation was reached. Data saturation was determined through iterative analysis of the interview transcripts. Once no new insights or themes were identified across successive interviews, data collection was concluded. The interviews lasted between 20 and 30 minutes. Participants were encouraged to share their perspectives freely, allowing for a comprehensive understanding of their experiences. The participants were informed that they might be contacted within one week of the first interview to clarify any ambiguities that might appear when reviewing the interview, but only one participant was contacted again and asked to clarify some of her statements. All interviews were conducted in Arabic for all generations with a total of 50 women including 17 mothers, 18 grandmothers, and 15 great-grandmothers.

Data analysis

The analysis was initiated by listening to the audio-recorded interviews to build an understanding of the raw material. Then, the interviews were transcribed verbatim by the RA. The transcriptions were then read and reread, by the PI, several times to get a sense of the whole. Lindgren, Lundman [15] steps for analyzing qualitative data, with a qualitative content analysis methodology, were then used with an inductive coding approach. The careful reading of the transcribed interviews led to the identification of meaning units that were translated into English. The meaning units were then coded, and the codes were compared regarding similarities and differences before creating subcategories that built the basis for category creation. All categories and subcategories were triangulated by two authors independently. This step was followed by a meeting to discuss the analysis methodology and the manifest and latent interpretation of the data [15]. Possible difficulties or obstacles met during the data analysis were also discussed before an agreement was reached on the final categories that represent the whole population and answer the aim of this study. Once a mutual agreement on the developed categories was reached, relevant citations in the interviews were decided and agreed on to confirm the findings and be presented in the results section.

Noteworthy, since the principal investigator [AK] is the only Arabic-speaking member of the research authors, she had the primary responsibility for the initial reading of the raw material and translation of the meaning-bearing units. These were subsequently analyzed and interpreted together with another team member, and finally critically reviewed by the third author.

Rigor

To ensure robustness, the research team extensively engaged with the data through repeated readings of participants' transcripts. To ensure data credibility, cross-checking was done with

the recorded data and the transcribed narratives, thereby minimizing the potential for individual biases. Cross-checking is a methodological approach used to enhance the trustworthiness of qualitative research findings. The method involves comparing the original audio recordings of interviews with their transcriptions to verify that the transcribed narratives accurately reflect what was said by the participants, thereby enhancing the credibility of the research findings ([16].

Results

The study included participants from three generations: mothers, grandmothers, and great-grandmothers. Participants were characterized by varying ages and socio-demographic backgrounds.

Generational perspectives on breastfeeding practices

The results are presented based on the mothers' (The mothers' experiences), grandmothers' (The grandmothers' experiences), and the great-grandmothers' (The great grandmothers' experiences) own unique stories where they shared similarities over the generations as well as experienced challenges. Although some of the identified categories were similar in all generations, there were some differences in practices, perceptions, and priorities related to breastfeeding. Table 2 provides an overview of the categories and subcategories.

The mothers' experiences

Proud to provide breastfeeding – a general unspoken rule, with exceptions. The participating mothers talked about how they perceive breastfeeding as a sign of motherhood and bonding, an expression of emotions that was regardless of the physical challenges they encountered.

Table 2. Generational-based experiences of breastfeeding practices – categories and sub-categories.

Generation	Categories	Sub-categories
**Mothers**	Proud to provide breastfeeding – a general unspoken rule, with exceptions	A threat to breastfeeding – work, social media, and formula
		Breastfeeding is a sign of motherhood and bonding
		Physical challenges were easily overcome
		Making the choice – to breastfeed
	Influencing factors – partner, family, and community	Workplace and lifestyle constraints – balancing responsibilities
		Advice to future generations
**Grandmothers**	Inherited practices	Including breastfeeding in daily life
		Customs and traditions for good or bad
	Religion and health – the child's right to breastfeeding	
	The spoiled generation versus the determined generation	Accomplished motherhood and the power of breastfeeding
		Priorities in life – baby's needs vs mother's physical appearance
**Great grandmothers**	Cultural and religious aspects – the instinct of caring	Culturally supportive systems – include the healthcare providers
		Knowledge – pave the way to continue breastfeeding
	The available mother – comparing now and then	New generations differ from us
		Adaption to different challenges

https://doi.org/10.1371/journal.pone.0319789.t002

A threat to breastfeeding – work, social media, and formula. Mothers shared experiences of how the availability of the formula and the mother's presence affected the choice between breastfeeding and formula. Some mothers narrated how the easiness of getting formula made the mothers choose to skip trying to breastfeed their babies, which they perceived affected the bonding between the mother and baby. The mothers meant that the current practices relied on perceiving formula as a problem-solving alternative and they tend to use it more liberally despite the awareness of its possible side effects and the importance of EBF.

Additionally, mothers talked about their generation's view of physical appearance and how it affected their and their babies' health, citing body image concerns as a significant impediment to EBF. They also meant that mothers of previous generations tend to breastfeed more than current generations, which was explained as a result of the social development and the changed circumstances that led mothers to choose the formula more nowadays compared to previous generations. The mothers reflected on the older generations' practices, meaning that they did not have alternatives to breastfeeding, the freedom to choose, or the commercial influences to affect their choices.

Breastfeeding is a sign of motherhood and bonding. The mothers underscored the decision to breastfeed was taken based on their previous experiences of breastfeeding the first children and the outcomes of this time's practices and that previous experiences could affect the determination to EBF. The mothers shared experiences of how breastfeeding gave them feelings of satisfaction, mental comfort, and a good mood. They perceived the value of motherhood, and that it is not completed unless breastfeeding the baby.

> "*Maternal instinct and the gift of these children are entrusted to us, so as long as I am able, I care for my child, so breastfeeding is among the caring practices that I give to my child.*" (M17)

Many of the participating mothers talked about instinctive motherhood and the gift of having children. They perceived breastfeeding as a natural process and the baby's right during the first two years of life. A mother expressed what breastfeeding meant for her motherhood as: "*Basically, you only feel the value of motherhood by breastfeeding from or the moment after giving birth.*" (M50).

Physical challenges were easily overcome. Some mothers described advice given by the older generations to consume complementary herbs such as fenugreek to stimulate lactation. They felt that this use made their overall breastfeeding experience easier, particularly when they experienced inadequate lactation and were concerned that their breastmilk might not be sufficient for their baby.

> "*It can be tiring for the mother, especially in the first few months. However, it also means that the mother is very close to her child and feels psychologically comfortable knowing that she is providing something useful that the child needs.*" (M17)

Some mothers talked about tolerating the physical challenges of breastfeeding for their own benefits. For example, they used physical appearance as a motivator for breastfeeding believing that breastfeeding is a natural weight control method.

The participating mothers narrated how persistent practice gave them skills. They witnessed how determination, a positive attitude towards breastfeeding, and presence during breastfeeding could all turn difficult beginnings into success. With persistent practices, mothers understood their children's needs and breastfeeding patterns, they said. The mothers

told stories about how they adhered to the baby's needs and were not neglecting the baby when not breastfeeding. They narrated about experiences of daily obstacles and how they managed to carry on with the breastfeeding task, expressing feelings of gratitude for their determination and strength to do something good for the baby which made the struggle worthwhile.

> "*The first thing is never to give formula as an option, always go natural. The second thing, if there is a struggle, then there is not only one solution for it, no, now there are 500 solutions, and even in health centers there are people who support one and there are programs that support breastfeeding. Try the first time, the second time, do not put it down.*" (M32)

Making the choice – to breastfeed. One of the practices experienced by mothers as successful was the continuity of breastfeeding. A mother described how she considered combining breastfeeding and formula, but experienced that EBF was stimulating lactation and the baby's acceptance of breastfeeding. Mothers shared the belief that with every child they breastfeed, they became more experienced and empowered in their decision-making which shaped their EBF practices.

Other mothers had an optimistic view of practicing EBF because of the health awareness campaigns, they meant. The immediate effect of health education was described by a mother providing her baby with a mix of breastfeeding and formula, but she decided to EBF once she heard the recommendations provided by a physician. Other mothers meant that not knowing what the best feeding alternative is, is not a valid reason for not breastfeeding. Practices shared by many mothers were taking responsibility for asking for advice, searching for information, and questioning the existing breastfeeding practices.

Influencing factors – partner, family, and community. Besides the self-determination, the mothers narrated about the irreplaceable support they received from their mothers in all from technical issues to motivation and encouragement. A mother talked about her experience of receiving support from the breastfeeding awareness nurse, and how the support of the nurse extended from the hospital stay to the first time in the home. Other mothers experienced healthcare providers as the first supportive persons in educating and motivating EBF, together with their own mothers, partners, female relatives, and close friends.

> "*My mother, my family, and my husband encouraged me (to breastfeed), and when we go to the health centers, there are girls there, health educators, who give advice and lectures, they always advise and guide.*" (M11)

The mothers said that they relied on their previous experiences of child feeding patterns, although they received different influential experiences from their community. A few mothers described the lack of support from family members, but meant that breastfeeding became a priority for every new child. They felt that when they were encouraged to prioritize breastfeeding before the formula, the decision was not difficult.

The mothers described experiences of how society perceives breastfeeding in different ways depending on the mothers' priorities; some would prioritize breastfeeding although they are employed, and some prefer formula alternatives although they are housewives, mainly to skip the struggles of breastfeeding during the night.

> "*In the second child, I had more experience in breastfeeding and how to store milk. This means that you will not be influenced easily, I mean by people who recommend formula, you will have more experience with your child.*" (M15)

Mothers mentioned frequently the perception that "Breastfeeding is a gift from God" which obligated them to breastfeed as long as they could. They mentioned the advice given by relatives to always provide breastfeeding as it was perceived as the baby's right and God's gift to the baby. In addition, mothers meant that for some people, breastfeeding was considered important, but not for others. They also reflected on the social transition with the increased economic capability to afford formula, which led to perceiving mothers EBF as less affluent. Mothers practicing EBF described it as a "cost-effective gift" (M17) and encouraged other mothers to use it.

Workplace and lifestyle constraints – balancing responsibilities. Mothers shared experiences of facing many challenges in combining their work with breastfeeding, including lack of time, work environment, and lack of experience and knowledge. They blamed work for neglecting the breastfeeding needs of mothers. Mothers reported that they did not have enough time in the work environment to express breastmilk, which was described as one of the main barriers to practicing EBF. Some mothers perceived their workplaces as non-promoter or non-breastfeeding friendly, as they lacked a separate or isolated place to breastfeed or express breastmilk. However, some mothers admitted neglecting breastfeeding because of their engagement in work, feeling that they did not give breastfeeding the importance it deserved.

> "*Society needs health awareness, we are in the age of transformation and rapid development, and easy solutions should be avoided when there are other healthier and more useful methods to be used. For example, conserving the breastmilk is better than using formula and this should be the focus of the health awareness campaigns.*" (M16)

On the other hand, the work environment was described by some mothers as a promoter or encourager of breastfeeding, as more experienced colleagues provided continuous encouragement and solutions to breastfeeding difficulties. Yet, some disparities between the private and public sectors were highlighted.

> "*Frankly, I hope that employees are considered regarding this point; sometimes, the workplace does not have a place designated for breastfeeding, and the same applies to breastfeeding hours. This means that we do not have time, we struggle to get time to express milk for the baby. We should be considered in this point. In private companies, there are breastfeeding hours, and even though we are employees in the health sector, and the health sector encourages (EBF) and everything, we, its employees, do not get that support.*" (M4)

Mothers talked about how they were aware of the side effects of formula milk, but felt they had no choice but to provide it when they were under pressure, busy, or had to leave the house for a few hours.

Advice to future generations. Experienced mothers imparted advice to current and future generations, advocating breastfeeding as the primary feeding choice. The current generation emphasized the main differences in breastfeeding practices between generations were related to nutritional habits. They meant that previous generations consumed more healthy food and, thus were able to produce enough breastmilk compared to current generations, who tend to consume fast foods more frequently and, thus have diminished lactation.

The grandmothers' experiences

The grandmothers' experiences were reflected in three main categories with two underlying subcategories to respective main categories (Table 2).

Inherited practices. Breastfeeding was perceived as an easy task by grandmothers who provided breastfeeding for their children. Some grandmothers reported that they did not receive any encouragement or support to provide breastfeeding, but they believed that their significant others would have noticed and possibly questioned their decision if they did not breastfeed. They also emphasized that the choice to breastfeed is dependent on the mother's knowledge and priorities, and therefore, they did not need any encouragement. Grandmothers said that they were convinced to breastfeed due to various reasons, and once they made the decision, there was no reason not to initiate breastfeeding.

> "*We take from the experience of the older ones, my mother always used to say when she heard my child shouting, "Drink thyme," and when she felt that I was uncomfortable, "Take the fenugreek", so we focused on these things and learned them, the passing on of experiences.*" (GM6)

Including breastfeeding in daily life. Grandmothers narrated situations in which they found that breastfeeding interfered with their daily activities, such as when they were tired, busy, or had guests. They described how they adapted to the situation and learned to manage breastfeeding despite these challenges, emphasizing that daily activities were never an excuse or a hindrance to their breastfeeding practices. They described also how they adjusted their breastfeeding practices to accommodate their work situations and the demands of their children's needs.

> "*It is true that it is difficult, because sometimes you are tired, exhausted, busy, or you have visitors (…), but it is possible to adapt to it. Tiresome, in general. I mean, breastfeeding is not comfortable, except for the non-working woman who knows that she is planning to be present all the time at home. I believe that it poses a great difficulty for the working woman.*" (GM6)

However, not only positive aspects were narrated in the interviews, but negative aspects were also mentioned, such as the perception that breastfeeding mothers were more limited due to the baby's dependency on them, which could be tiring for the mother.

Customs and traditions for good or bad. Grandmothers' decision to breastfeed their own children was often based on their experiences with breastfeeding in their previous families. At the same time, grandmothers living in compound homes with their husbands' families felt that social judgment and expectations limited their healthy breastfeeding practices. They meant that traditions and customs of breastfeeding practices were expected to be followed, regardless of the challenges mothers faced. In a try to understand the demands of older generations, grandmothers believed that they would become like their mothers when encouraging their own daughters to breastfeed, although with more understanding of the physiological and mental changes that exist between different individuals.

Breastfeeding was described as a general, unspoken rule that mothers knew was the right thing to do since their own mothers and grandmothers did the same and encouraged them to do so as well. Some grandmothers called the advice given by their own mothers about breastfeeding "inherited experiences" and believed in their usefulness based on successful outcomes for their own mothers. Yet, grandmothers who could not meet the unwritten social expectations of breastfeeding their babies, despite trying all recommended herbs and complementary foods, felt frustrated and thought that this frustration could be one of the reasons for limited milk production. Mothers felt that the matter of breastfeeding was more a matter of following cultural norms, customs, and traditions, as well as following their own instinct to nourish the baby, rather than listening to their own body and its ability to produce milk. They also noted

clear differences between the older generations and the current ones in terms of priorities and household duties.

> "*Our first mothers' breastfeeding experiences were different, as they lacked education and faced various challenges. They made a great effort, managing all household chores, taking care of the children, and working in agriculture and with livestock (…) the baby was immediately followed by another baby and the period of breastfeeding for them was a short period. This time is different, with working women and more help at home.*" (GM46)

Grandmothers felt that the increased focus on work outside the home in today's generations hindered breastfeeding. However, they meant that available support and methods for expressing breastmilk and conserving it made breastfeeding possible even for working mothers, regardless of their employment status.

Religion and health – the child's right to breastfeeding

Mothers found religious beliefs to be a driving force for providing breastfeeding. They also chose breastfeeding to protect their children from potential future diseases. Mothers who used formula experienced feelings of guilt, as they believed that breastfeeding was the superior method for nourishing their children and used these feelings as a motivator for breastfeeding.

Mothers acknowledged the differences in breastfeeding practices between generations, with older generations relying more on natural processes. They believed that the natural processes resulted in healthier children, less prone to infections, and stronger bodies. Mothers believed that breastmilk was a gift from God to their children, and therefore, they should not prohibit the child from getting access to it: "*We should not be cheap in breastfeeding our children*" (GM20) or from benefitting from it: "*Breastfeeding is the nutritional value provided by the mother to the child.*" (GM45). The participating grandmothers meant that they were knowledgeable about the benefits of breastfeeding and the recommendations of Quranic verses on breastfeeding for two consecutive years which was experienced as a strong motivator for EBF.

Their belief that a child had the right to be breastfed for a period of two years was expressed as "*There is no in-between alternative*" (GM41). Mothers who were unable to breastfeed, despite genuine efforts and seeking support experienced feelings of disappointment believing it is the child's right to get a safe, clean, and better nutritional alternative which is breastfeeding, according to the participating grandmothers.

The narratives of the participants revealed that healthcare providers played a significant supportive and educative role in promoting EBF. However, the mothers meant that there were instances when they failed to provide a healthy environment to attach the baby to the mother and initiate breastfeeding. Some mothers reflected on the collective role of society, the support system, and healthcare professionals in encouraging the healthy practice of EBF, and thus leading to more compliance. Women who were surrounded by encouraging people, receiving empowering messages, and support from partners and other significant relatives expressed that they felt more willing to breastfeed. In addition, the encouragement of the mother was reflected in almost all interviews. In addition, positive influences and encouragement from partners and other significant relatives were reported to be strong motivators for breastfeeding. The role of a supportive father was highlighted, with some fathers being described as demanding of breastfeeding, while others were supportive of the choice.

> "*My husband encouraged me to breastfeed. He completely refuses to give formula, and also my mother encourages me, and my neighbors and friends encourage mostly natural ways.*" (GM37)

Grandmothers emphasized the availability and variability of information sources in the Omani society, including social media, which gave them the possibility to prepare, plan, and make information-based decisions. However, changed literacy patterns were described as the main reason for the changed breastfeeding practices. For example, after being informed on the proper way of expressing and storing breastmilk, mothers chose this practice meaning that it is a way of providing long-time breastfeeding.

The spoiled generation versus the determined generation. The interviewed grandmothers shared a perception of the current generation as lazy and not caring for their children, regardless of their occupational status. Some were even accused of running from their responsibility and letting the feeding responsibility fall to the nanny. The older generations were referred to as the encouraging generation who succeeded in their breastfeeding and passed their experiences to the current generations.

The perception of grandmothers in general, was that today's generations prefer formula for invalid reasons, such as prioritizing their physical appearance and their lazy attitudes towards breastfeeding. The lifestyle and attitudes of the current generation were perceived as the responsible factors for the current breastfeeding practices.

Grandmothers narrated the experience of today's mothers. They mentioned that the formula was not available all the time, and mothers of older generations had to adjust the baby's food if their lactation was limited, while mothers of today are more experienced in all available replacements.

> "*Now you know spoiled women who are so pretentious that they don't want to breastfeed their children anyway, and they don't give them time and they don't want to get up at night to breastfeed them.*" (GM47)

Mothers in today's generation were perceived as neither convinced nor committed to initiating or continuing breastfeeding. The interviewed mothers highlighted possible reasons for this lack of commitment, including the mental state of the mother, social situation, lack of support, and potential complications or illnesses. They gave the advice of "*Do not compare yourself with others*" (GM42) to master the breastfeeding challenges faced by current generations.

Accomplished motherhood and the power of breastfeeding. Accomplished motherhood and the power of breastfeeding are closely intertwined. The benefits of breastfeeding, mental preparedness, and commitment to breastfeeding are essential factors for successful breastfeeding practices.

Breastfeeding was perceived as a way to spend more time with the child, providing a good feeling to the mother, and allowing her to choose the most suitable feeding method for her baby. Mothers meant that women needed to be mentally prepared for the challenges of breastfeeding and appreciated the freedom to make their own choices.

> "*I spend more time with my baby when I breastfeed her.*" (GM15)

Breastfeeding was seen as a source of love, affection, and care, providing the opportunity to bond with the baby, socialize with them, and offer the love and affection they deserved. Mothers expressed pride in being able to provide breastfeeding for their children, despite the challenges they faced.

The grandmothers' narratives reflected how the responsibility shifted from the current generations' mothers to au pairs or close relatives to care for the baby, while the mother worked which led to significant consequences for breastfeeding practices among current generations.

As one of the older generations, they perceived themselves as women with healthier eating habits, which positively influenced their lactation and consequently their breastfeeding practices.

The experience of more frequent breastfeeding mothers showed that the number of breastfeeding times positively contributed to successful breastfeeding. Being convinced and committed to breastfeeding was crucial for overcoming the challenges grandmothers faced.

Priorities in life – baby's needs vs mother's physical appearance. The grandmothers expressed the belief that mothers should prioritize their baby's health by breastfeeding naturally, emphasizing the importance of this choice for the child's future well-being. They also highlighted the impact of body image issues, the influence of social media and celebrities, and the challenges faced by working mothers in maintaining breastfeeding practices.

Grandmothers expressed concerns about the current generation's lack of knowledge about the impact of breastfeeding on body shape and changes, as well as the shift in priorities from the baby's needs to the mother's physical appearance. They also discussed the difficulties faced by working mothers in balancing their professional and maternal responsibilities, which often led to insufficient time for bonding and providing other aspects of breastfeeding.

> "*But life has priorities, which is more important? My health, or the breast size for a woman, or the health of my son, whom I may cause diseases, whose immunity will be low, and he will be vulnerable to diseases. Priorities, the mother should be more educated on this issue*." (GGM39)

The narratives also revealed a need for more awareness and education to motivate mothers to prioritize breastfeeding, especially in the face of societal influences and the challenges of modern motherhood. The grandmothers emphasized the importance of understanding the consequences of prioritizing physical appearance over breastfeeding and the need for greater support for working mothers to maintain breastfeeding practices. Participants from older generations viewed this trend as a lack of seriousness about breastfeeding and a misplaced set of priorities.

The great-grandmothers' experiences

The great-grandmothers' narratives resulted in two main categories with two underlying subcategories per respective main category.

Cultural and religious aspects – the instinct of caring. The great-grandmothers revealed the influence of cultural and religious beliefs on breastfeeding practices. Mothers from older generations described how not breastfeeding was seen as a betrayal of the trust given to them by God, and that babies were perceived as gifts as well as breastfeeding adjusted to serve the needs of the baby through the first two years of its life. The ability to breastfeed was seen as a blessing, and mothers had the main responsibility to deliver this gift to their babies, not deprive them of it.

> "*First of all, this is one of their rights. It is a right mentioned in the Holy Qur'an, so the child must receive the first of his rights.*" (GGM45)

Mothers also discussed the instinct of caring, emphasizing that breastfeeding and caring for the baby are built into human nature. They believed that mothers are programmed to care for their babies and should prioritize them before work. The narratives also revealed that the length of the breastfeeding period was a concern for some mothers, as breastfeeding for two consecutive years made the baby more dependent and it was difficult to stop breastfeeding afterward.

"*The reason (for breastfeeding) is that it is a matter of customs, traditions and innate nature.*" (GGM43)

The participants also discussed the impact of modern facilities on breastfeeding practices, highlighting how previous generations had to take their children with them wherever they went because they did not have the facilities to express and conserve breastmilk in fridges. Breastfeeding was described as a joyful process/task, bringing feelings of happiness and satisfaction to mothers from older generations.

Culturally supportive systems – include the healthcare providers. The narratives of the great-grandmothers shed light on the multifaceted influences on breastfeeding practices, including the role of health educators, supportive information, self-motivation, and the impact of medical professionals' recommendations. The great-grandmothers described positively how health awareness educators specializing in breastfeeding were instrumental in overcoming initial obstacles during the early days in the hospital. Supportive information from leaflets and TV was also cited as influential in convincing mothers to choose EBF. Learning from previous generations, listening to their advice, observing behaviors, and confirming knowledge through reading were mentioned by the great-grandmother as strategies for improving breastfeeding practices in the current generation.

The great-grandmothers' narratives also revealed the complex interplay between self-motivation, cultural expectations, external influences, and medical recommendations that also might be wrong. Some mothers felt forced to provide formula due to concerns about the potential harm of breastfeeding during pregnancy, while others expressed regret for using the formula, attributing it to the notable side effects and their trust in medical professionals' recommendations.

"*In order for him not to lose weight, the doctors and nurses gave him formula, and when I went home, I was told that if there is not enough breastmilk, give him formula...and so I did.*" (GGM23)

This trust sometimes led to a lack of knowledge and feelings of guilt, as exemplified by a mother who was influenced by others to avoid breastfeeding due to concerns about inheriting thalassemia. Additionally, the impact of cesarean sections on breastfeeding practices was mentioned, with some mothers perceiving the use of formula as an adverse outcome of cesarean delivery.

Knowledge – pave the way to continue breastfeeding. Experiences were reflected about the impact of witnessing the side effects of formula in other children, the ease of the breastfeeding process, and the long-term effects of breastfeeding as motivators for continuing to breastfeed. The narratives highlighted the importance of maternal awareness of the benefits of breastfeeding, including its use as a contraceptive method and its natural, preventive, and protective properties. Mothers from older generations shared their experiences of using breastfeeding as a contraception method.

"*I gave my first children formula and kept getting pregnant. The after-following ones, I breastfed them naturally and I did not get pregnant. Once I discovered that, I kept breastfeeding my children without any addition of formula.*" (GGM28)

The available mother – comparing now and then. The discourse surrounding breastfeeding practices unveils contrasting perspectives between generations, shedding light on the evolving dynamics of motherhood. Great-grandmothers expressed concerns over what

they perceive as a shift in priorities among contemporary mothers, citing reasons such as personal appearance, work commitments, and a lack of health literacy as factors influencing the preference for formula over EBF. The sentiment of tenderness and responsiveness associated with breastfeeding, cherished by the participating great-grandmothers, stands in stark contrast to the perceived nonchalance and reluctance of the current generation towards this essential aspect of caregiving, according to them.

On the other hand, the adaptation to challenges in breastfeeding showcases a nuanced approach adopted by the great-grandmothers. They shared experiences of deep commitment to providing breastmilk, despite obstacles like work responsibilities, highlighting the value placed on the benefits of breastfeeding. They described using creative strategies, such as feeding with a spoon to sustain breastfeeding while working, underscoring their resilience and dedication in navigating these challenges.

The great-grandmothers underscored the importance of intergenerational dialogue to stress early initiation of breastfeeding, emphasizing the significance of bonding and responsiveness to the infant's cues for a successful breastfeeding journey. They meant that while differences in practices and perceptions exist, the shared goal of nurturing and providing the best for the baby remains a common thread, bridging the generational gap in the realm of breastfeeding practices.

New generations differ from us. Participants from older generations described the current generation as "spoiled" and "egoistic," emphasizing a prioritization of personal needs, particularly rest and sleep, over attending to the immediate needs of the baby. This marked a departure from the practices of older generations, who provided breastfeeding promptly in response to the baby's cues.

The intellectual transition of the current generations was identified as a primary factor contributing to the perceived lack of interest and priority in breastfeeding. Mothers were seen as shifting their focus from the needs of their children to prioritizing personal physical appearance, attributing the decline in breastfeeding enthusiasm to this transition.

> "*But nowadays, girls - I mean, this generation - feel that it is easier to give their children formula milk because they are afraid of losing their figure and having their breasts sag, so they prefer not to breastfeed.*" (GGM36)

Stubbornness, comfort, laziness, and a reluctance to heed advice were characteristics ascribed to the current generations. One of the participating great-grandmothers described the contemporary generation as mothers "*lacking interest and are careless*" (GGM36) and even called them "*lazy girls*" (GGM12). Mothers from the older generations expressed concern over the perceived non-adherence of the new generation to their babies' needs. They used strong language, describing the act of not breastfeeding when the baby required it as a form of "*torture*" (GGM18).

Furthermore, nonchalance, work commitments, and reliance on nannies were described as reasons why contemporary mothers were believed to prefer formula over breastfeeding. The great grandmothers meant that the current generation's apologies for not breastfeeding were often linked to circumstances such as work, travel, or illness, contrasting with the religious recommendations. Participants expressed a perception of the current generation's lack of interest and carelessness towards breastfeeding. Older generations recalled prioritizing the "*breastfeeding hour*" (GGM36) and making professional sacrifices for breastfeeding, whereas contemporary mothers often provided formula without a compelling reason such as work.

The lack of health literacy regarding breastfeeding was acknowledged, emphasizing the need for awareness about its benefits.

"*However, some of them feel that even when they are at home, they can give their children formula milk, they say that their breastmilk is not enough. This means that they do not have the literacy or awareness of the benefits of breastfeeding.*" (GGM33)

Economic development, the availability of formula, and a focus on physical appearance were identified as key reasons for the contemporary preference for formula over EBF. Even mothers from older generations acknowledged this shift meaning that the current generation differs from older ones.

Breastfeeding was perceived as a sign of mothers' tenderness by older generations, who felt that their generation exhibited more responsiveness, availability, and love by providing breastfeeding promptly in response to the baby's needs. The advice to compensate for absence by breastfeeding for the rest of the day, as given by the older generations, was contrasted with the observation that contemporary mothers often chose formula due to fatigue after a long working day, *"They don't listen to our advises"* (GGM5).

Great-grandmothers experienced resistance to the advice given by older generations, with the perception that the new generation follows convenience rather than the baby's needs. The marketing of formula, the broad assortment available, and competition among manufacturers were blamed by the older generation for influencing contemporary breastfeeding practices.

Adaption to different challenges. The availability of breastmilk and the priority given to breastfeeding emerged as major motivators among the older generation. Mothers expressed a deep sense of commitment to providing their babies with the benefits of breastmilk, considering it a fundamental aspect of caregiving. The participants also discussed the challenges of balancing work and breastfeeding, with some mothers feeling hindered by their work responsibilities. The participants underscored the importance of experiences of breastfeeding as the greatest motivator for mothers. Many mothers narrated how they used the formula as a help to strengthen breastfeeding and meet the baby's demands and some mothers turned to formula as a supportive measure, aiming to compensate for perceived challenges such as inadequate lactation, insufficient weight gain in the baby, or time constraints preventing frequent breastfeeding. This nuanced approach highlighted the pragmatic strategies employed by mothers to navigate breastfeeding challenges.

Participants shared examples of EBF, even in situations where mothers were working and needed to express breastmilk. In a creative adaptation, mothers would feed their babies with a spoon instead of a bottle, showcasing the flexibility and commitment to sustaining breastfeeding despite external demands.

This reflected a value placed on responsiveness and sensitivity to the baby's cues. Observations were made regarding babies refusing formula, leading mothers to adhere to the baby's needs and continue breastfeeding for the entire recommended period of two years. This illustrated the dynamic interaction between mothers and babies, emphasizing the importance of responsiveness to the infant's signals.

Mothers described the act of breastfeeding as a "*clean method*" (GGM22), conveying a sense of freedom and availability when providing their babies with breastmilk. This sentiment reflected the positive experiences associated with breastfeeding, beyond its nutritional benefits.

Drawing from their experiences, mothers from the older generation advised the current generation to initiate breastfeeding promptly. This initiation was seen as a key factor contributing to a smoother and more successful breastfeeding journey, emphasizing the importance of early bonding between mother and child.

Discussion

The study aimed to delve into the exclusive breastfeeding practices among three generations of mothers in Oman, presenting a unique opportunity to explore practices spanning multiple generations in a country undergoing political and economic transformations [17]. The findings highlight a complex interplay of factors influencing breastfeeding practices across generations. The three generations' experiences and practices are illustrated in Fig 1. The figure highlights that mothers take pride in their breastfeeding practices, while grandmothers view their experiences as inherited traditions. Great-grandmothers, on the other hand, adapted their practices to overcome challenges encountered during their breastfeeding journeys. Notably, both mothers and grandmothers shared common experiences related to the prioritization of breastfeeding. Additionally, religious aspects of breastfeeding were emphasized by both grandmothers and great-grandmothers, while the availability of mothers was a shared experience recounted by both great-grandmothers and mothers. A significant overlapping theme across all generations is the perception of breastfeeding as an essential aspect of motherhood.

Younger generations of mothers view breastfeeding as a vital expression of motherhood and bonding, but encounter challenges such as career demands, social media influences, and body image concerns. They mistakenly believe breastfeeding negatively affects their appearance, impacting their choice between breastfeeding and formula. Recent studies indicate that certain women view breastfeeding positively concerning body image, recognizing the strength and functionality of their bodies during this period, and expressing pride in their bodies for nourishing their infants despite acknowledging physical changes [18]. Yet, other research findings indicate that women often associate breastfeeding with significant changes in their bodies, leading to mixed feelings about their physical appearance. For instance, women who reported dissatisfaction with their body image were less likely to plan for breastfeeding or continued breastfeeding for shorter durations compared to those who felt positive about their appearance [19]. This correlation suggests that body image directly impacts a mother's decision to breastfeed, as negative perceptions can lead to early cessation of breastfeeding due to feelings of unattractiveness or discomfort with physical changes [20, 21]. Furthermore, contemporary generations of mothers face heightened scrutiny regarding their parenting choices, including feeding practices. Societal expectations often equate "good motherhood" with breastfeeding, creating a moral imperative that pressures mothers to conform [22]. Moreover, social media plays a significant role in shaping body image perceptions among new mothers. The portrayal of idealized post-pregnancy bodies can lead to unrealistic expectations and increased dissatisfaction with one's own body during the breastfeeding phase [23]. This phenomenon is particularly concerning as it may deter mothers from embracing the functional

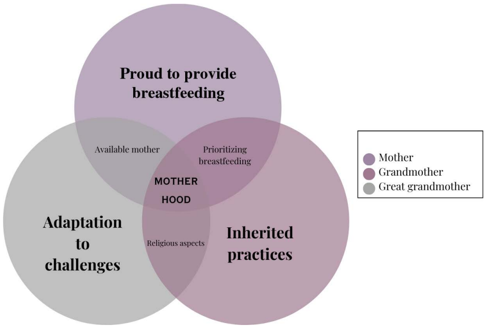

Fig 1. Conceptual model of breastfeeding experiences and practices across generations in Oman.

https://doi.org/10.1371/journal.pone.0319789.g001

aspects of their bodies during this transformative period. Therefore, support networks for new mothers, comprehensive education on the benefits of breastfeeding, and promotion of media literacy initiatives to encourage critical engagement with social media portrayals of motherhood and body image could all contribute to healthy breastfeeding practices. In addition, by fostering an environment that prioritizes positive body image and supports breastfeeding practices, we can help contemporary generations of mothers navigate the complexities of motherhood while promoting healthier feeding choices for their infants.

The participating grandmothers expressed practices following professional advice such as early initiation, the need for the presence of the women to care for and be with her child, and using the experiences received from other women and their own children. Breastfeeding gave them a sense of freedom and intimacy with their children which they hope to pass on to the younger generations of women in Oman. This intimate connection can be interpreted as a biological instinct and a necessity for a deeply emotional experience that enhances mothers' confidence and nurtures the mother-child relationship. Research indicates that breastfeeding promotes physical closeness, which is essential for emotional bonding and attachment ([24]. In the context of Oman, where cultural values emphasize family and community, the act of breastfeeding can symbolize a mother's commitment to nurturing and raising her children in a supportive environment, thereby instilling these values in younger generations [7]. Although Omani women are among the populations with the highest rates of ever-breastfeeding [25], reinforcing the positive impact of the Omani cultural values regarding EBF could be an effective strategy for sustaining these healthy practices. Therefore, it is recommended that the healthcare sector and public health policymakers take action to strengthen initiatives and promotional campaigns aimed at maintaining the healthy practices of EBF among contemporary generations, following the guidance and advice of the older generations.

Statistics indicate that 48,4% of children are breastfed beyond 2 years of age, with 71.1% initiating breastfeeding in the first hour after birth [26], which is an important factor in prolonged breastfeeding. Older generations always prioritized breastfeeding [26]. Similar findings were reported by the women in our study, across generations. This could be due to the support women had from religion and society, which gave many of them the advantage of being together with their smaller children at home. Grandmothers reflect on inherited practices, customs, and traditions that inform their breastfeeding decisions, highlighting the significance of societal expectations and religious beliefs. All generations underscore the crucial role of support from partners, family, and healthcare providers in promoting exclusive breastfeeding for the first six months and beyond, aligning with the multifaceted approach advocated by UNICEF [25]. Strategies to optimize breastfeeding and surmount breastfeeding barriers are crucial, including policies guaranteeing parental leave, the right to breastfeed in the workplace, and restrictions on the marketing of breastmilk substitutes. Within healthcare facilities, mothers need information and support to breastfeed immediately after birth, and beyond [27]. Cultivating positive social norms that encourage breastfeeding, including in public spaces, empowers mothers to breastfeed [28]. In communities, support from trained counsellors and peers, including other mothers and family members plays a key role, and also the support of men, husbands, and partners cannot be underestimated [25]. Some generational changes have been reported in the Gulf countries as a result of the social development in the region [29]. This might explain the unchanged EBF patterns and practices for the last two decades with a low rate of 23.2% as reported in the Oman National Nutrition Survey 2017 [30]. The advice to future generations of mothers in this study is to emphasize the significance of making breastfeeding a primary choice, underscoring the evolving nutritional habits and societal influences impacting breastfeeding practices [31]. However, this is not possible

without the legal support of societal structures, such as sufficient parental leave [32], making it possible for women to fully take part in society, as well as family life.

The great-grandmothers in this study appear to have lived in a supportive, breastfeeding-positive society. In addition, the great-grandmothers emphasized the importance of knowledge in making informed choices rather than being influenced by commercial factors which aligns with Riad, 2023 [33] who meant that when mothers have the appropriate knowledge, they are empowered to make informed choices about breastfeeding, rather than being swayed by commercial factors. It is of utter importance that policymakers and stakeholders help and support breastfeeding mothers and act according to the International Code of Marketing of breastmilk substitutes [34], …"*that it should not be marked or distributed in ways that may interfere with protection and promotion of breastfeeding…*" (p. 10) as well as supporting the Baby Friendly Hospitals Initiative. In 1999, all marked hospitals in Oman were thus certified [35] as Baby Friendly, according to WHO and UNICEF. In addition, monitoring mechanisms for the implementation of the code, 'Omani Code for Marketing of Breastmilk Substitutes' are being strengthened [5]. Furthermore, great-grandmothers mentioned the importance of the mothers' availability and prioritisation of breastfeeding before work-related commitments and duties in sustaining EBF practices. A possible explanation of the current study's findings could be that older generations started their motherhood journey at an early age, experiencing their first childbirth at an average age of 15.85 years compared to 20.9 years for current generations [36], which might have delayed women's opportunities of taking part of the labor and educational force in society. Oman pays special attention to women and their contribution to the Omani society [37]. According to a report from the Arab Center, women in Oman are making progress in entrepreneurship, leadership, politics, and education, with opportunities for participation in the workforce and legal advances [17,38]. At the same time, they are expected to fulfil motherhood and the responsibility of breastfeeding and many of the women in this study were facing many obstacles. Studies that have been conducted on breastfeeding in Oman have identified that the majority of infants of six-month-old babies are exposed to formula feeding very early [6,8,39,40]. To promote a breastfeeding-friendly society, it is essential to consider gender equality, shifting the responsibility from individual women to society as a whole [41]. According to recent changes in Oman's labor law, maternity leave has been increased from 50 to 98 calendar days, with no limit on the number of times maternity leave may be taken [42]. Female employees returning to work from maternity leave are entitled to one hour per day of paid breastfeeding leave. Additionally, female employees may request up to one year of unpaid leave following the birth of their child [42]. Despite recent improvements in maternity leave policies in Oman, which now include one hour per day of paid breastfeeding leave and the option for up to one year of unpaid leave following childbirth, these changes still fall short of the recommended six months of exclusive breastfeeding [43]. Additionally, the impact of social media and increased marketing of formula, combined with limited support and information, contributes to low rates of exclusive breastfeeding in Oman and globally [28,31].

The older generations of mothers in this study highlighted the religious and cultural support they received for breastfeeding, viewing it as a mandatory aspect of good motherhood. Despite facing difficulties such as milk shortages and wounds, they remained deeply committed to breastfeeding, reflecting the high value placed on its benefits. Similarly, contemporary Omani mothers demonstrate resilience and a strong belief in the power of breastfeeding, overcoming challenges despite limited knowledge and difficult circumstances [7].

Research implications and recommendations

This study offers valuable insights into the multifaceted dynamics influencing breastfeeding practices among Omani mothers across generations. The findings underscore the need

for targeted interventions and policy initiatives to support and promote breastfeeding in Oman. Policymakers could consider implementing comprehensive support programs that address societal, cultural, and economic factors affecting breastfeeding choices. Educational campaigns aimed at dispelling myths and misconceptions about breastfeeding, particularly regarding its impact on maternal appearance and career opportunities, could be beneficial. Furthermore, healthcare providers should prioritize breastfeeding education and support, ensuring that mothers receive accurate information and assistance throughout their breastfeeding journey.

Study limitations

Despite its contributions, this study has a few limitations. Firstly, the qualitative nature of the research limits its transferability, as the findings may not be representative of all Omani mothers' experiences. Additionally, the retrospective nature of the study may have introduced recall bias, impacting the accuracy of participants' recollections. Furthermore, participating women showed different abilities to articulate their experiences which can lead to variations in the depth of information provided, and possibly affect the overall analysis and findings. While the intention was to create an open environment for participants to share their stories, the inconsistency in expression may introduce challenges in synthesizing and comparing findings across interviews [44], potentially affecting the study's conclusions and credibility of the results. Nevertheless, no challenges were experienced by the research team and the fact that all women were given the possibility to express themselves freely added a significant value to the analysis process. In addition, the fairly high number of interviews conducted was considered a relevant compensation for this limitation. Finally, cultural and societal factors unique to Oman may limit the transferability of the findings to other contexts. However, since Oman shares many cultural perspectives and practices with other countries in the region and the Islamic world, some of our findings might be transferable to other populations in the mentioned contexts.

Conclusions

The key findings of this study highlight the perception of breastfeeding as a sign of motherhood and bonding, fulfilling emotional, religious, cultural, and social expectations, despite the challenges mothers face. While all generations perceived EBF as a child's right, the current generations are seen as less interested due to concerns about body image, influenced by advertisements, and engaged in work outside the home. They also struggle with prioritization, placing body image, work duties, and social commitments above breastfeeding. Despite the differences in practices and perceptions across generations, the common goal of nurturing and providing the best for the baby remains a unifying theme, helping to bridge the generational gap in breastfeeding practices. Therefore, fostering a breastfeeding-supportive environment requires multifaceted strategies that address societal, workplace, and individual factors. Given the potential impact of working life and life outside the home as aggravating factors, it is imperative for societal institutions to contribute to the development of policies such as extended parental leave. By achieving an EBF target rate of $>90\%$ by 2025, societal barriers to EBF can be addressed, and women can be supported in their motivations to EBF.

Moreover, investing in community education programs and healthcare provider training can further promote breastfeeding as the optimal feeding choice for infants, thereby contributing to the health and well-being of future generations. Continued research and evaluation are essential to inform the development of effective breastfeeding support initiatives and ensure their sustained impact on maternal and child health outcomes.

Supporting information

S1 Data. COREQ (COnsolidated criteria for REporting Qualitative research) checklist is attached.
(DOCX)

Acknowledgments

We would like to acknowledge Ms. Nawal Al Amri for her valuable assistance throughout the data collection process.

All authors approved the submitted version and agree to be personally accountable for their own contributions and to ensure that questions related to the accuracy or integrity of any part of the work are appropriately investigated, resolved, and the resolution documented in the literature.

Author contributions

Conceptualization: Atika Khalaf.

Formal analysis: Atika Khalaf, Rebecca Mathew, Pernilla Ny.

Funding acquisition: Atika Khalaf.

Methodology: Atika Khalaf.

Project administration: Atika Khalaf.

Writing – original draft: Atika Khalaf, Pernilla Ny.

Writing – review & editing: Rebecca Mathew, Pernilla Ny.

References

1. WHO, UNICEF. Global Breastfeeding Scorecard 2022: Protecting Breastfeeding through further investments and policy actions. 2022. Report No.: WHO/HEP/NFS/22.6. Available from: https://www.who.int/publications/i/item/WHO-HEP-NFS-22.6
2. UNICEF, WHO. Global Breastfeeding Scorecard 2022: Protecting Breastfeeding through bold National actions during the Covid-19 pandemic and beyond. 2021 Oct. Report No.: WHO/HEP/NFS/21.45. Available from: https://www.globalbreastfeedingcollective.org/media/1591/file
3. Rollins NC, Bhandari N, Hajeebhoy N, Horton S, Lutter CK, Martines JC, et al. Why invest, and what it will take to improve breastfeeding practices?. Lancet. 2016;387(10017):491–504. https://doi.org/10.1016/S0140-6736(15)01044-2 PMID: 26869576
4. WHO U. Nutrition Landscape Information System (NLiS) Country Profile - Oman. 2023 Updated. Available from: https://apps.who.int/nutrition/landscape/global-monitoring-framework?ISO=omn
5. Al-Ghannami S, Atwood S. National Nutrition Strategy: Strategic Study 2014–2050. Dep Nutr Minist Health Muscat Oman. 2014.
6. Al Barwani S, Hodges E, Crandell J, Thoyre S, Knafl K, Sullivan C. Predicting infant feeding intention and intensity in Oman using a path analysis approach. East Mediterr Health J. 2023;29(5):324–34. https://doi.org/10.26719/emhj.23.057 PMID: 37306169
7. Al-Marzouqi Z. Perception of Omani Women of Exclusive Breastfeeding: A Grounded Theory Study. The University of Manchester (United Kingdom). 2020. Available from: https://research.manchester.ac.uk/en/studentTheses/perception-of-omani-women-of-exclusive-breastfeeding-a-grounded-t
8. Al-Ghannami S, Al-Mamari S, Chekaraou D, Abla C, Al-Ghmmari I, Al-Ajmi A, et al. Exclusive Breastfeeding: Barrier analysis amongst Omani mothers. Sultan Qaboos Univ Med J. 2023;23(2):158–67. https://doi.org/10.18295/squmj.5.2022.038 PMID: 37377837
9. Dukuzumuremyi JPC, Acheampong K, Abesig J, Luo J. Knowledge, attitude, and practice of exclusive breastfeeding among mothers in East Africa: a systematic review. Int Breastfeed J. 2020;15(1):70. https://doi.org/10.1186/s13006-020-00313-9 PMID: 32795377
10. Kaluba C, Nakazwe KC, Sichimba F. From grandmother to first-time mother: A qualitative study of prenatal and postnatal maternal socialization in Lusaka, Zambia. J Hum Behav Soc Environ. 2023;34(5):768–82. https://doi.org/10.1080/10911359.2023.2190361

11. Chai Y, Nandi A, Heymann J. Does extending the duration of legislated paid maternity leave improve breastfeeding practices? Evidence from 38 low-income and middle-income countries. BMJ Glob Health. 2018;3(5):e001032. https://doi.org/10.1136/bmjgh-2018-001032 PMID: 30364395

12. Negrin KA, Slaughter SE, Dahlke S, Olson J. Successful recruitment to qualitative research: a critical reflection. Int J Qual Methods. 2022;21. https://doi.org/10.1177/16094069221119576

13. Oman by region. In: Foreign Ministry [Internet]. Available from: https://www.fm.gov.om/about-oman/state/oman-by-region/

14. Parsa-Parsi RW. The International Code of Medical Ethics of the World Medical Association. JAMA. 202210.1001/jama.2022.19697. https://doi.org/10.1001/jama.2022.19697 PMID: 36227623

15. Lindgren B-M, Lundman B, Graneheim UH. Abstraction and interpretation during the qualitative content analysis process. Int J Nurs Stud. 2020;108103632. https://doi.org/10.1016/j.ijnurstu.2020.103632 PMID: 32505813

16. Halcomb EJ, Davidson PM. Is verbatim transcription of interview data always necessary?. Appl Nurs Res. 2006;19(1):38–42. https://doi.org/10.1016/j.apnr.2005.06.001 PMID: 16455440

17. Liloia A. The Role of Women in Oman's Social and Economic Progress. Arab Center Washington D.C; 2022. Available from: https://arabcenterdc.org/resource/the-role-of-women-in-omans-social-and-economic-progress/

18. Primo CC, Ferreira CI, Soares J da S, Muri LAC, Lima E de FA, Brandão MAG. Body image of women during breastfeeding: analysis supported by nursing theory. Rev Gaucha Enferm. 2023;44e20220051. https://doi.org/10.1590/1983-1447.2023.20220051.en PMID: 37283431

19. Morley-Hewitt AG, Owen AL. A systematic review examining the association between female body image and the intention, initiation and duration of post-partum infant feeding methods (breastfeeding vs bottle-feeding). J Health Psychol. 2020;25(2):207–26. https://doi.org/10.1177/1359105319833744 PMID: 30873876

20. Acheampong AK, Abukari AS. Nurses' and midwives' perspectives on how the pursuit for the "perfect" body image affects their own breastfeeding practices: a qualitative study in Ghana. Int Breastfeed J. 2021;16(1):74. https://doi.org/10.1186/s13006-021-00421-0 PMID: 34565392

21. Kapa HM, Litteral JL, Keim SA, Jackson JL, Schofield KA, Crerand CE. Body Image Dissatisfaction, Breastfeeding Experiences, and Self-Efficacy in Postpartum Women with and Without Eating Disorder Symptoms. J Hum Lact. 2022;38(4):633–43. https://doi.org/10.1177/08903344221076529 PMID: 35139671

22. Severinsen C, Neely E, Hutson R. Resisting stigma: the role of online communities in young mothers' successful breastfeeding. Int Breastfeed J. 2024;19(1):17. https://doi.org/10.1186/s13006-024-00626-z PMID: 38448916

23. Tang L, Tiggemann M, Haines J. #Fitmom: an experimental investigation of the effect of social media on body dissatisfaction and eating and physical activity intentions, attitudes, and behaviours among postpartum mothers. BMC Pregnancy Childbirth. 2022;22(1):766. https://doi.org/10.1186/s12884-022-05089-w PMID: 36224523

24. Linde K, Lehnig F, Nagl M, Kersting A. The association between breastfeeding and attachment: A systematic review. Midwifery. 2020;81:102592. https://doi.org/10.1016/j.midw.2019.102592 PMID: 31830673

25. UNICEF. Breastfeeding: A mother's gift, for every child. New York: Nutrition Section, Programme Division, UNICEF; 2018 pp. 1–20. Available from: https://www.unicef.org/media/48046/file/UNICEF_Breastfeeding_A_Mothers_Gift_for_Every_Child.pdf

26. Al-Jawaldeh A, Abul-Fadl A, Al-Sumaie M, AbuNyayan A, Al-Ghannami S. Status of breastfeeding in the Arabian Peninsula and Gulf countries of the Eastern Mediterranean region. MFC Egypt J Breastfeed. 2019;1561–74. https://doi.org/10.6084/m9.figshare.8940119

27. Walsh A, Pieterse P, Mishra N, Chirwa E, Chikalipo M, Msowoya C, et al. Improving breastfeeding support through the implementation of the Baby-Friendly Hospital and Community Initiatives: a scoping review. Int Breastfeed J. 2023;18(1):22. https://doi.org/10.1186/s13006-023-00556-2 PMID: 37061737

28. Dykes C, Ny P, Hauck YL, Kuliukas L, Gallagher L, Brady V, et al. Women's perceptions of factors needed to encourage a culture of public breastfeeding: a cross-sectional study in Sweden, Ireland and Australia. Int Breastfeed J. 2023;18(1):49. https://doi.org/10.1186/s13006-023-00583-z PMID: 37658398

29. Rahman MM, Al-Azm A. Social Change and Transformation in the Gulf Region. In: Rahman MM, Al-Azm A, editors. Social Change in the Gulf Region: Multidisciplinary Perspectives. Singapore: Springer Nature; 2023. pp. 1–14. https://doi.org/10.1007/978-981-19-7796-1_1

30. UNICEF Oman M. Oman national nutrition survey 2017 | UNICEF Oman. 2018 Jan. Available from: https://www.unicef.org/oman/reports/oman-national-nutrition-survey-2017
31. Prentice AM. Breastfeeding in the Modern World. Ann Nutr Metab. 2022;78(2):29–38. https://doi.org/10.1159/000524354 PMID: 35679837
32. USBC. Existing Legislation. In: US Breastfeeding Committee [Internet]. [cited 30 Apr 2024]. Available from: https://www.usbreastfeeding.org/existing-legislation.html
33. Riad J. Knowledge Management & Decision Making: How Leaders Can Make More Informed Decisions. J Educ Leadersh Action. 2023;8(3):. https://doi.org/10.62608/2164-1102.1133
34. WHO. International code of marketing of breast-milk substitutes. World Health Organization; 1981. Available from: https://www.who.int/publications/i/item/9241541601
35. Sinani MA. Breastfeeding in Oman-The way forward. Oman Med J. 2008;23(4):236–40. PMID: 22334834
36. Missal B. Gulf Arab women's transition to motherhood. J Cult Divers. 2013;20(4):170–6. PMID: 24575592
37. Amzat IH, Al-Ani WTK, Ismail OH, Al Omairi T. Women's empowerment and its effect on community development in Oman: predictive model and indicators for best practices. Community, Work & Family. 2017;22(3):338–56. https://doi.org/10.1080/13668803.2017.1367274
38. The World Bank. Labor force participation rate, female (% of female population ages 15+) (modeled ILO estimate) - Oman. The World Bank; 2023. Available from: https://data.worldbank.org/indicator/SL.TLF.CACT.FE.ZS?locations=OM
39. Al-Nuaimi N, Katende G, Arulappan J. Breastfeeding Trends and Determinants: Implications and recommendations for Gulf Cooperation Council countries. Sultan Qaboos Univ Med J. 2017;17(2):e155–61. https://doi.org/10.18295/squmj.2016.17.02.004 PMID: 28690886
40. Al Maamari S, Al Shammakhi S, Alghamari I, Jabbour J, Al-Jawaldeh A. Young Children Feeding Practices: An Update from the Sultanate of Oman. Children (Basel). 2021;8(9):818. https://doi.org/10.3390/children8090818 PMID: 34572250
41. Pérez-Escamilla R, Tomori C, Hernández-Cordero S, Baker P, Barros AJD, Bégin F, et al. Breastfeeding: crucially important, but increasingly challenged in a market-driven world. Lancet. 2023;401(10375):472–85. https://doi.org/10.1016/S0140-6736(22)01932-8 PMID: 36764313
42. Lockton O. Oman expands parental leave entitlements and introduces an Omanization requirement. 2023.
43. Omer-Salim A. Breastfeeding and Work: A Balancing Act. 2023. Available from: https://www.un.org/en/un-chronicle/breastfeeding-and-work-balancing-act#:~:text=Once%20a%20mother%20returns%20to,as%20long%20as%20she%20chooses
44. Wellard S, McKenna L. Turning tapes into text: issues surrounding the transcription of interviews. Contemp Nurse. 2001;11(2–3):180–6. https://doi.org/10.5172/conu.11.2-3.180 PMID: 11924614
